# Complete Response in Salivary Duct Carcinoma Ex Pleomorphic Adenoma With Upfront Combination of Trastuzumab and Chemo-Hormonal Therapy

**DOI:** 10.7759/cureus.82742

**Published:** 2025-04-21

**Authors:** Ajay Gupta, Aadithya Lakshmi Narayanan, Vikas Kashyap, Surabhi Gupta

**Affiliations:** 1 Medical Oncology, Indraprastha Apollo Hospitals, New Delhi, IND; 2 Histopathology and Cytopathology, Indraprastha Apollo Hospitals, New Delhi, IND; 3 Radiodignosis, Indraprastha Apollo Hospitals, New Delhi, IND

**Keywords:** androgen receptor, carcinoma ex pleomorphic adenoma, combined androgen blockade, cxpa, her2, salivary duct carcinoma, trastuzumab

## Abstract

Salivary duct carcinoma (SDC) is one of the rarest malignancies. It can arise de novo or from pre-existing pleomorphic adenoma (carcinoma ex pleomorphic adenoma - CXPA) and can be very aggressive.

Here, we present one such case of a 48-year-old male with relapsed, widely invasive SDC ex pleomorphic adenoma of the parotid that was locally advanced with intracranial extension that was refractory to chemo-radiation. Immunohistochemistry confirmed positivity for human epidermal growth factor receptor-2 (HER2) and androgen receptor (AR). He was started on a combination of trastuzumab, Combined Androgen Blockade (CAB) with leuprolide and bicalutamide, and docetaxel. Follow-up imaging at three months and nine months from treatment initiation demonstrated a complete response, ongoing at 17 months at follow-up. The upfront combination of anti-HER2 therapy, CAB, and chemotherapy resulted in a durable complete response in an aggressive disease.

The result obtained from our experience provides valuable insight into the management of a rare and treatment-resistant entity, where large prospective studies without heterogeneity are not possible.

## Introduction

Salivary gland carcinomas (SGCs) comprise about 8% of all head and neck cancers. The salivary duct carcinoma (SDC) histotype constitutes 1% to 4% of all SGCs [1]. Carcinoma ex pleomorphic adenoma (CXPA), stemming from the malignant transformation of pre-existing pleomorphic adenoma (PA) of the parotid gland is a rare and highly aggressive form. Pleomorphic adenomas are benign salivary gland tumors, most commonly involving the parotid gland. Histologically, it's a mixed tumor with epithelial and myoepithelial components. Malignant transformation occurs due to genetic instability in recurrent or long-standing PAs, with peak incidence in the 7th decade of life, predominantly in male patients [2].

CXPA can be of any histological subtype, but salivary duct carcinoma is the commonest [3], with about one-third of SDCs arising from a pre-existing PA [4]. Other histological types include adenocarcinoma, not otherwise specified (NOS), myoepithelial carcinoma, mucoepidermoid carcinoma, and adenosquamous carcinoma [2].

The disease typically presents as a longstanding palpable mass in the parotid area with recent rapid growth and pain. Pre-operative diagnosis is largely based on history and clinical examination. The disease can be intracapsular, minimally invasive (<1.5 mm beyond the capsule), or widely invasive (>1.5 mm beyond the capsule). High-grade tumors demonstrate perineural and vascular invasion and hence tend to be very aggressive [2]. Older age, extra-capsular invasion, higher tumour grade, and lymph node involvement correlate with poorer prognosis, and the 5-year overall survival (OS) is 25%−65% [3]. 

While surgery and radiation therapy have a role in locoregional disease, systemic therapy is employed in advanced disease, with a molecularly informed approach to treatment playing an important role. However, standard guidelines specific to CXPA treatment have not been established due to a lack of large-scale prospective trials, given the rarity of this disease entity.

Screening for human epidermal growth factor receptor-2 (HER2) and androgen receptor (AR) positivity has been widely adopted. AR is a nuclear hormone receptor that binds androgens and is expressed in ~90% of SDCs and drives tumor growth. HER2 is a transmembrane tyrosine kinase receptor involved in cell proliferation and is overexpressed or amplified in ~29%-46% of SDCs, especially high-grade tumors. Epidermal growth factor receptor (EGFR) is also frequently overexpressed in SDC, however, actionable mutations are rare. Gene mutations in phosphoinositide 3-kinase (PIK3CA), p53 tumor suppressor protein (TP53), and Harvey Rat Sarcoma viral oncogene (HRAS) can also be screened for in these patients. Loss of mismatch repair (MMR) proteins leads to microsatellite instability (MSI), but this is quite rare in SDC [5].

Studies have shown co-expression of HER2 and AR in the SDC histotype [4], and sequential use of anti-HER2 therapies (AHT) and androgen deprivation therapies (ADT) has been tried in a few cases as single agents, with or without concurrent cytotoxic chemotherapy, in a few cases [6-9]. However, there is no data in the literature on the combined upfront use of AHT and ADT.

We present the following report of a patient with relapsed, refractory, widely invasive salivary duct carcinoma ex pleomorphic adenoma of the parotid gland, with poor prognostic factors of high-grade transformation, infiltrative pattern, perineural involvement, and nodal metastasis, in whom we have obtained a continuing complete response (CR) of 17 months duration, with anti-HER2 targeting (AHT) and combined androgen blockade (CAB), along with chemotherapy.

## Case presentation

**Figure 1:**
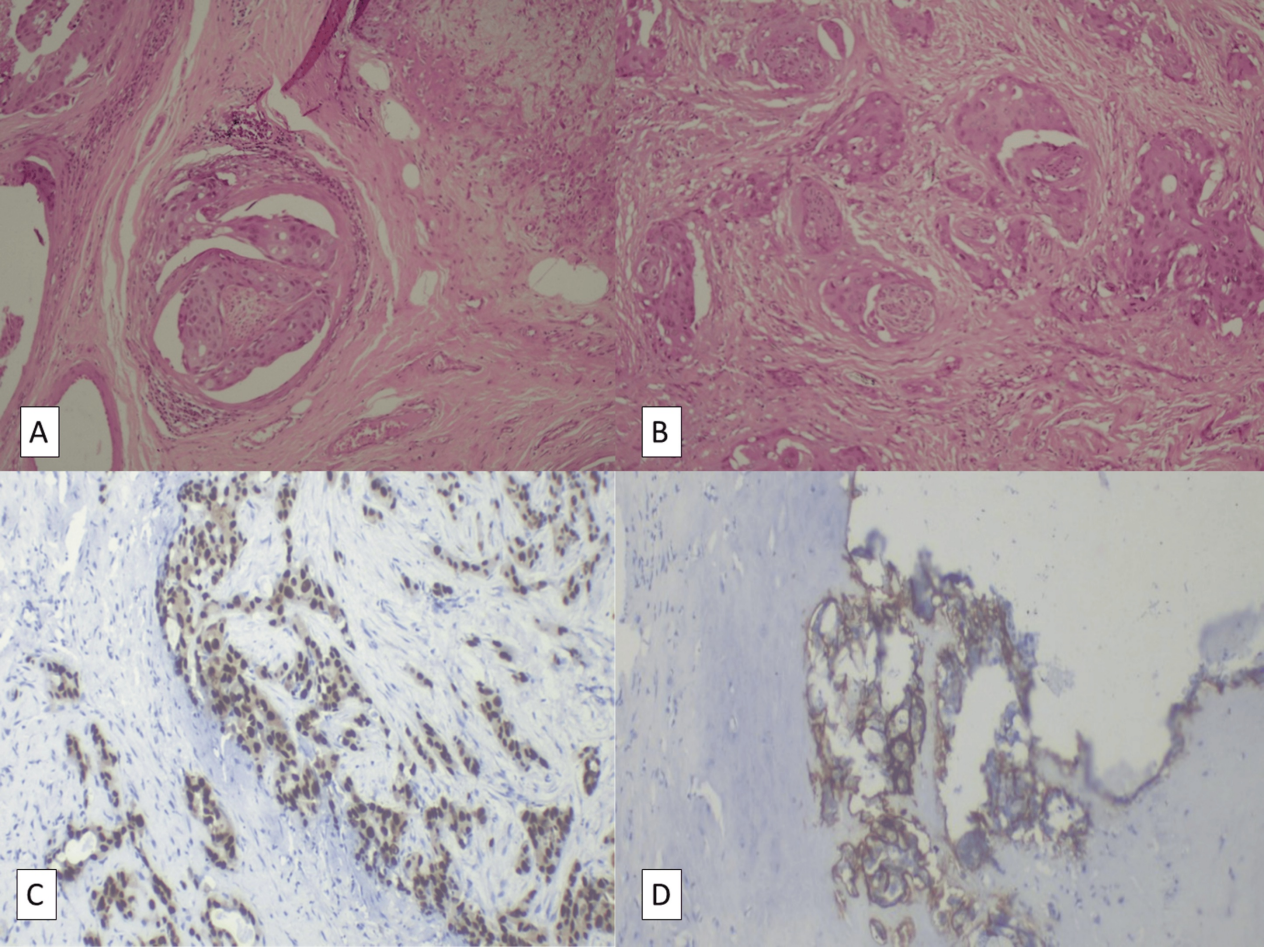
Histopathology A. (10x) Right upper edge shows residual pleomorphic adenoma, and the rest shows foci of high-grade carcinoma, demonstrating malignant transformation in the pre-existing benign tumor (carcinoma ex pleomorphic adenoma) (28-03-2022) B. (10x) Solid nests of tumor in carcinomatous foci with perineural invasion, suggesting the highly aggressive nature of the disease (28-03-2022) C. Immunohistochemical staining shows 90% tumor cells stained with strong intensity for androgen receptors (AR), suggesting that anti-androgen therapy will be useful against these malignant cells (10-10-2023) D. Immunohistochemical staining shows human epidermal growth factor receptor-2 (HER2) positivity (3+), suggesting that anti-HER2 therapy (trastuzumab) will be useful against these malignant cells (10-10-2023)

A 48-year-old, non-smoking, non-alcoholic, male from Northern India, with no co-morbidities, was diagnosed as a case of pleomorphic adenoma in 2000, for which he underwent local resection. In 2012, the patient had a disease recurrence but did not take treatment. In 2022, he started to experience right-sided facial weakness and pain, for which he was evaluated. Fine needle aspiration cytology was suggestive of poorly differentiated carcinoma. He then underwent a right total parotidectomy with right supraomohyoid neck dissection in March 2022. Post-operative histopathological examination demonstrated a unifocal tumor (3.5 cm x 3.2 cm x 2 cm), which involved the entire gland. It showed high-grade transformation and poor circumscription with an infiltrative pattern and brisk mitosis (Figure 1A). Lympho-vascular space invasion and significant perineural invasion were identified (Figure 1B). Six out of 19 nodes at level II were found to have deposits with extranodal extension. The final report described an invasive carcinoma ex pleomorphic adenoma of the salivary duct carcinoma subtype, with pathological staging: pT3N2b (as per pathologic tumor node metastasis (pTNM) staging, American Joint Committee on Cancer, 8th edition).

Subsequently, in April-May 2022, he received adjuvant radiotherapy to the tumor bed and right neck nodal basin (33 fractions of 66 Gray), with six cycles of weekly concurrent cisplatin therapy (~40 mg/m2). There were no significant therapy-related adverse effects.

In July 2023, the disease relapsed. He was restarted on radiation therapy, targeting the right infratemporal fossa and the intracranial component (16 fractions of 38.3 Gray). Towards the end of radiation therapy, the patient started to experience double vision, which later progressed to complete loss of vision in the right eye and markedly diminished vision in the left eye.

**Figure 2:**
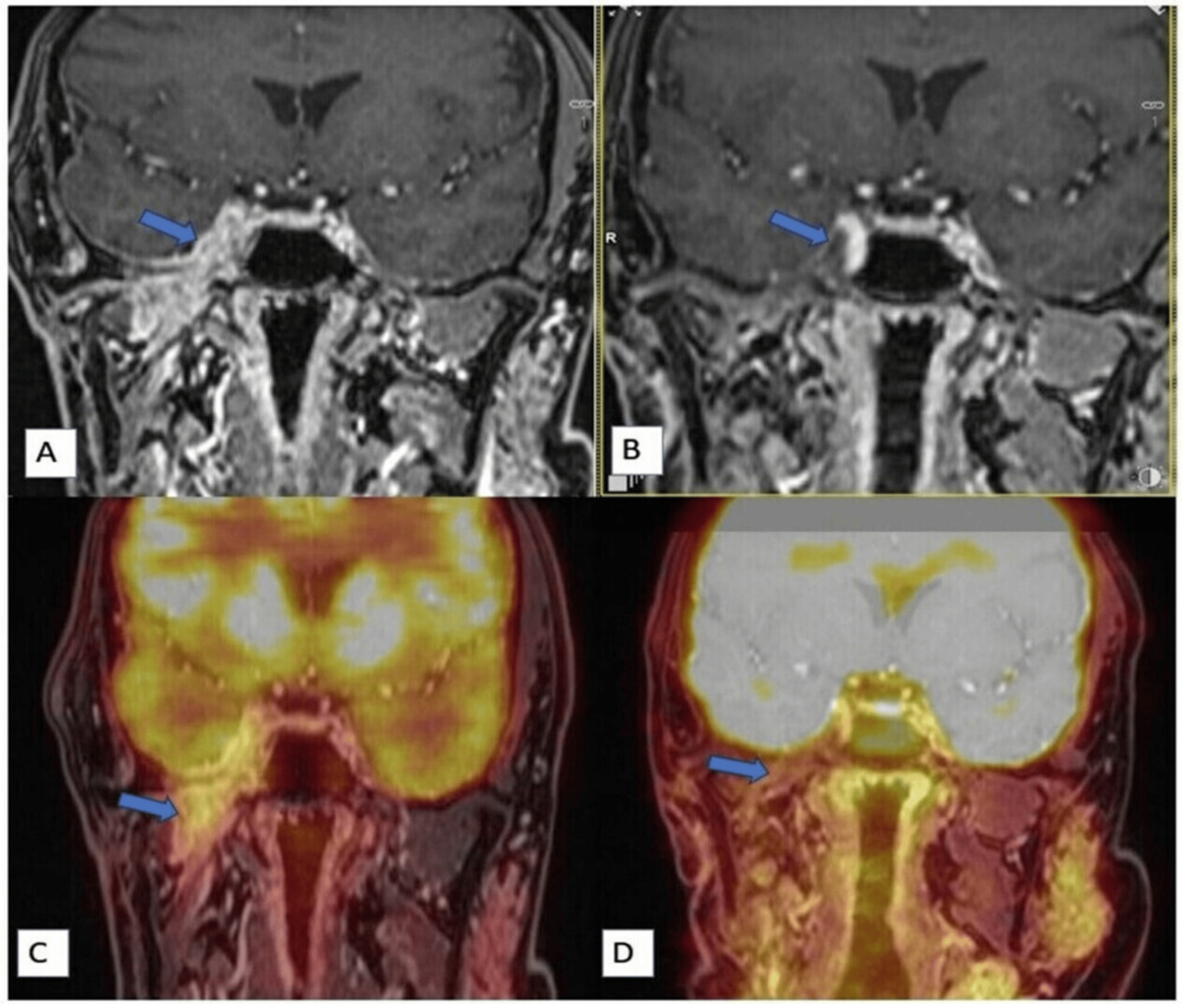
Regression on radiology A, C. (Sept 2023, before the combination regimen started) Post-contrast T1 coronal image (A) and corresponding fused PET-MR image (C) show 18F-fluorodeoxyglucose (FDG)-avid enhancing thickening (arrow) near the skull base with intracranial extension in the right Meckel's cave and cavernous sinus region. B, D. (Aug 2024, with treatment): Post-contrast T1 coronal image (B) and corresponding fused PET-MR image (D) show a complete response.

An evaluation with positron emission tomography with magnetic resonance imaging (PET-MRI) (September 2023) revealed progression of disease in the right infratemporal fossa and right masticator space, involving the right masseter and the lateral and medial pterygoids, with right mastoiditis. There was also evidence of intracranial extension into the cavernous sinus region and the Meckel’s cave, with an extradural component in the right medial temporal lobe, indenting the underlying parenchyma. There was partial encasement of the right cavernous carotid with disease extending up to the right optic canal, with involvement of the superior rectus muscle (Figure 2A). At the time of writing this report (October 2023), immunohistochemical analysis of the original parotid resection demonstrated strong ADR positivity and HER2 overexpression (Figure 1C, 1D) but was negative for programmed death-ligand 1 (PD-L1), epidermal growth factor receptor (EGFR), and microsatellite instability (MSI).

Considering the high risk of complete loss of vision, an aggressive treatment approach was adopted and, from October 2023, the patient was started on trastuzumab (loading dose of 8 mg/kg, followed by 6 mg/kg) three weekly and CAB, with leuprolide 22.5 mg every three months and daily oral bicalutamide 100 mg, alongside chemotherapy with docetaxel (~70 mg/m2) every three weeks for six cycles. This was followed by maintenance trastuzumab, leuprolide, and bicalutamide.

PET-MRI in February 2024 (three months after starting our regimen) showed marked regression in the bulk of the disease, with overall complete regression in metabolic avidity. PET-MRI in August 2024 (9 months after starting our regimen) showed further regression in the bulk of the disease, with a sustained complete metabolic response (Figure 2B, 2D).

The patient reported a marked improvement in vision in his left eye and now continues to be disease-free for 17 months since the start of this regimen.

## Discussion

SDC shares pathological and immunohistochemical similarities with ductal carcinoma of the breast and prostate cancer [4, 10]. It has also been shown that SDC has AR pathways and ADT-resistance mechanisms similar to prostate carcinoma [10]. This is further confirmed by the fact that SDC (both de novo and CXPA) has overexpression of AR and HER2, approximately 78-96% and 29%-46%, respectively, with co-expression reported in 30% of the cases worldwide [4].

There is a paucity of studies focussing on CXPA as a separate entity, pushing us to extrapolate data available for SDCs.

Positive outcomes have been shown with AHT-based regimens in both SDC de novo and SDC ex pleomorphic adenoma. In the largest prospective study to date, from Japan, involving 57 SDC cases, trastuzumab plus docetaxel demonstrated an overall response rate (ORR) of 70.2% (CR in 14%), and median progression-free survival (PFS) of 8.9 months and OS of 39.7 months [1].

The largest prospective trial, also from Japan, which incorporated CAB in 36 cases of SGC, the majority of which were SDC type, demonstrated ORR of 42% (CR 11%), median PFS of 8.8 months, and OS of 30.5 months. Interestingly, it was also observed that CAB had improved response rates compared to single-agent ADT [11].

In cases with co-expression of AR and HER2, the literature is not clear on what strategy to adopt. Indirect comparisons, however, indicate the superiority of trastuzumab-based therapies as the first-line treatment [4]. Multiple studies have reported partial response (PR) or stable disease with AHT and ADT used sequentially, but no large-scale studies have been carried out. There are no studies describing the combined upfront use of ADT, AHT, and chemotherapy. Some studies that employed sequential use of these therapies, in cases with co-expression of AR and HER2, have been summarised in Table 1.

**Table 1:** Strategies used in the treatment of SDC co-expressing HER2 and AR AR: androgen receptor; ADT: androgen deprivation therapies; HER2: human epidermal growth factor receptor-2; CAB: combined androgen blockade; PR: partial response; PFS: progression-free survival

Study [ref]	No. of cases	AR and HER2 Status	Systemic therapy used	Outcomes
Van Boxtel et al., 2017 [6]	n=2	AR+, HER 2+	Case 1: 1^st^ line: ADT, led to progression, 2^nd^ line: trastuzumab + pertuzumab + docetaxel	PR, PFS 17 months
			Case 2: 1^st^ line: trastuzumab + pertuzumab + docetaxel , 2^nd^ line: ADT, 3^rd^ line: trastuzumab emtansine	PR, PFS 4 months
S.D Jeong at al., 2020 [7]	n=1	AR+, HER 2+	Carboplatin + leuprolide + bicalutamide	PR, PFS 8 months
Čavka et al., 2023 [8]	n=1	AR+, HER 2+	1^st^ line: cyclophosphamide, doxorubicin, and cisplatin, 2^nd^ line: goserelin and bicalutamide (CAB), 3^rd^ line: trastuzumab + paclitaxel, 4^th^ line: trastuzumab +vinorelbine, 5^th^ line: capecitabine	Death after 5 years from diagnosis
Rösch M et al., 2021 [9]	n=1	AR+, HER 2+	Leuprolide + bicalutamide, chemotherapy, immunotherapy, and trastuzumab had been used prior to starting androgen blockade	PR, PFS 14 months, continuing regression of intracranial metastases

Combining different available treatment modalities to achieve optimal outcomes has been the cornerstone of cancer treatment. In HER2-positive breast cancer with co-existing hormone receptor positivity, trastuzumab with chemo-hormonal therapy is an accepted treatment combination. It has also been noted that their combined antitumor activity was greater than any of the agents given alone [12].

Similarly, in prostate cancer, the benefits of combination chemo-hormonal therapies are well-established by the CHAARTED trial [13].

Drawing an inference from data on breast and prostate, adding CAB upfront to AHT-based chemotherapy might significantly improve CR and PFS in SDC because of the immunohistochemical similarities.

## Conclusions

We achieved CR, extending over 17 months and ongoing, in our patient who had widely invasive, relapsed, refractory disease.

Literature highlights the superiority of molecularly informed therapy compared to single-agent regimens. Multiple studies have reported the combination use of cytotoxic chemotherapy and anti-HER2 therapies like trastuzumab, pertuzumab, and trastuzumab emtansine in aggressive cases of SDC to achieve improved PFS and OS in most patients, and the use of combined androgen blockade as another option in case of refractory disease. Very few have realised the therapeutic potential of targeting the co-expression of HER2 and AR. Even in studies that identified both receptors, treatments targeting these receptors have only been used sequentially, with some benefit.

It has been demonstrated that targeting multiple receptors concurrently provided improved outcomes in prostate (chemo-hormonal) and breast cancer (targeted therapy with cytotoxic chemotherapy with or without hormonal therapy). We chose to apply the same principle in this case, where the disease was aggressive and severely affecting the quality of life of our patient.

Given the significant incidence of SDCs with co-expression of AR and HER2 (30%), targeting both receptors upfront may be associated with better patient outcomes.

The result obtained from our experience provides valuable insight into the management of a rare, treatment-resistant entity with combination therapy, where large prospective studies without heterogeneity are difficult.
